# 达拉非尼联合曲美替尼治疗*BRAF *p.L485_T488delinsF突变转移性非小细胞肺癌1例

**DOI:** 10.3779/j.issn.1009-3419.2025.106.25

**Published:** 2025-08-20

**Authors:** Yunfei WANG, Wen ZHAO, Chuang YANG, Rongyu ZHANG, Chengjun WANG, Chunyan HAN, Jisheng LI

**Affiliations:** ^1^250012 济南，山东大学齐鲁医院肿瘤内科（王云菲，赵文，杨闯，张荣雨，王程君，李际盛）; ^1^Department of Medical Oncology, Qilu Hospital of Shandong University, Jinan 250012, China; ^2^250011 济南，山东中医药大学附属医院肿瘤放疗科（韩春燕）; ^2^Department of Radiotherapy Oncology, Affiliated Hospital of Shandong University of Traditional Chinese Medicine, Jinan 250011, China

**Keywords:** 肺肿瘤, *BRAF*突变, 达拉非尼, 曲美替尼, 靶向治疗, Lung neoplasms, *BRAF *mutation, Dabrafenib, Trametinib, Targeted therapy

## Abstract

鼠类肉瘤病毒癌基因同源物B（v-Rafmurine sarcoma viral oncogene homolog B, *BRAF*）是最重要的原癌基因之一，是丝裂原活化蛋白激酶（mitogen-activated protein kinase, MAPK）/细胞外信号调节激酶（extracellular signal-regulated kinase, ERK）信号通路的关键调节因子。*BRAF*突变在非小细胞肺癌（non-small cell lung cancer, NSCLC）患者中的发生率为1.5%-5.5%，其中*BRAF* V600突变占所有*BRAF*突变的30%-50%，*BRAF* V600E是最常见的突变类型。目前，达拉非尼联合曲美替尼已被美国国立综合癌症网络（National Comprehensive Cancer Network, NCCN）、欧洲肿瘤内科学会（European Society of Medical Oncology, ESMO）、中国临床肿瘤学会（Chinese Society of Clinical Oncology, CSCO）等多个国内外指南推荐作为*BRAF* V600突变NSCLC的一线治疗选择。然而，针对*BRAF*非V600突变，目前尚无明确的靶向治疗推荐。尽管个案报道提示达拉非尼联合曲美替尼对部分*BRAF*非V600突变患者可能有效，但由于病例数量有限且缺乏大样本临床试验数据，其疗效和安全性仍需进一步验证。本文报道了1例经达拉非尼联合曲美替尼治疗有效的罕见*BRAF*插入缺失突变（*BRAF* p.L485_T488delinsF）的NSCLC病例，旨在提高临床医生对该类罕见突变病例的认识，并为未来治疗策略的探索提供参考。

## Abstract (en)

The v-Raf murine sarcoma viral oncogene homolog B (*BRAF*) gene is one of the most critical proto-oncogenes and functions as a key regulator in the mitogen-activated protein kinase (MAPK)/extracellular signal-regulated kinase (ERK) signaling pathway. The incidence of *BRAF *mutations in non-small cell lung cancer (NSCLC) patients ranges from 1.5% to 5.5%, with *BRAF* V600 mutations accounting for approximately 30%-50% of all *BRAF* mutations, among which *BRAF *V600E represents the most prevalent mutation type. Currently, the combination of Dabrafenib and Trametinib has been recommended as first-line therapy for *BRAF* V600-mutant NSCLC by multiple domestic and international guidelines including National Comprehensive Cancer Network (NCCN), European Society of Medical Oncology (ESMO), and Chinese Society of Clinical Oncology (CSCO). However, there are no clear targeted treatment recommendations for *BRAF* non-V600 mutations. Although case reports suggest that Dabrafenib combined with Trametinib may be effective for patients with *BRAF *non-V600 mutations, the efficacy and safety require further validation due to limited sample size and lack of large-scale clinical trial data. This article reports a case of NSCLC with a rare *BRAF* insertion and deletion mutation that responded well to the treatment of Dabrafenib in combination with Trametinib, aiming to enhance clinicians' understanding of such NSCLC cases with extremely rare mutation and provide a reference for future treatment strategies.

肺癌是我国发病率和死亡率最高的恶性肿瘤，其中非小细胞肺癌（non-small cell lung cancer, NSCLC）占所有肺癌的80%以上^[1]^。随着分子靶向治疗的快速发展，驱动基因阳性 NSCLC患者获得了全新的治疗选择，但临床实践中仍面临着诸多挑战。基因检测技术的进步和应用让越来越多的罕见驱动突变（突变发生率<5%）被发现并成为潜在的治疗靶点，其中鼠类肉瘤病毒癌基因同源物B（v-Raf murine sarcoma viral oncogene homolog B, *BRAF*）基因作为丝裂原活化蛋白激酶（mitogen-activated protein kinase, MAPK）/细胞外信号调节激酶（extracellular signal-regulated kinase, ERK）信号通路的关键调控因子，被认为是重要的致癌驱动因素，其靶向治疗也取得显著进展。*BRAF*基因突变在NSCLC中的发生率为1.5%-5.5%，在所有的*BRAF*突变中，*BRAF* V600突变占30%-50%，尤以V600E最为常见^[2,3]^。针对该类患者，*BRAF*与丝裂原活化细胞外信号调节激酶（mitogen-activated extracellular signal-regulated kinase, MEK）抑制剂联合治疗可以显著改善预后，如达拉非尼联合曲美替尼方案在多项临床试验中展现出优异的客观缓解率（objective response rate, ORR）和无进展生存期（progression-free survival, PFS）。然而，非V600突变患者却面临治疗困境，*BRAF*非V600突变（包括II类/III类突变）因激酶活性依赖RAS（rat sarcoma）或二聚化特性，对现有BRAF抑制剂反应不佳，目前尚无明确靶向治疗推荐。尽管个案研究^[4,5]^提示双靶方案对部分*BRAF*非V600突变可能有效，但大样本临床证据仍缺乏。本文报道了1例携带罕见*BRAF* p.L485_T488delinsF插入缺失突变的转移性NSCLC患者临床资料，旨在为此类特殊变异NSCLC患者的诊断和治疗提供参考。

## 1 临床资料

患者女，68岁，2024年3月因“咳嗽咳痰半月余”就诊于山东大学齐鲁医院，体格检查示右肺呼吸音减弱，可闻及散在湿啰音；左肺呼吸音清晰；浅表淋巴结未触及肿大。电子计算机断层扫描（computed tomography, CT）检查示右肺门及纵隔占位，首先考虑为恶性肿瘤；双肺多发结节，考虑转移；右肺阻塞性肺炎、阻塞性肺不张；双肺多发炎症、纤维灶；左肺肺大疱；双侧胸腔及心包积液。正电子发射断层显像/CT（positron emission tomography/CT, PET/CT）示右肺恶性病变并双肺、右侧顶枕部、胰尾、左侧肾上腺及左侧腰背部皮下结节转移可能性大（图1）。颅脑磁共振成像（magnetic resonance imaging, MRI）示：右侧顶枕叶占位，考虑转移瘤（图2A-C）。支气管镜检查示右肺中间段支气管远端可见新生物附着，右肺中下叶管腔扭曲、狭窄；右上叶支气管黏膜充血、浸润，管腔尚通畅。活检病理示（右下叶开口）恶性肿瘤，免疫组化均无表达，考虑为NSCLC，非特指类型（non-small cell carcinoma, not otherwise specified, NSCC-NOS）。免疫组化：甲状腺转录因子-1（thyroid transcription factor-1, TTF-1）（-），细胞角蛋白7（cytokeratin 7, CK7）（-），细胞角蛋白5/6（cytokeratin 5/6, CK5/6）（-），p63（-），p40（-），突触素（synaptophysin, Syn）（-），嗜铬粒蛋白A（chromogranin A, CgA）（-），CD56（-），新天冬氨酸蛋白酶A（NapsinA）（-），胰岛素瘤相关蛋白1（insulinoma-associated protein 1, INSM1）（-），Ki-67阳性率为30%（图3）。家族史：其兄因食管癌去世。结合影像和病理结果，诊断为右肺NSCC-NOS伴脑、肺、肾上腺、胰腺、皮下转移（cT4N3M1c2，IVB期）。

**图1:**
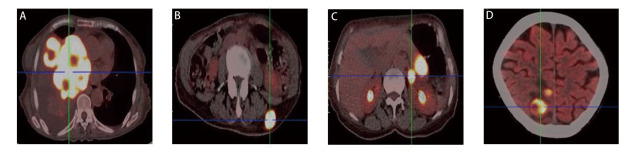
基线影像学检查。 A：右肺门及纵隔占位；B：皮下转移结节；C：胰尾、肾上腺转移；D：右侧顶枕部转移。

**图2:**
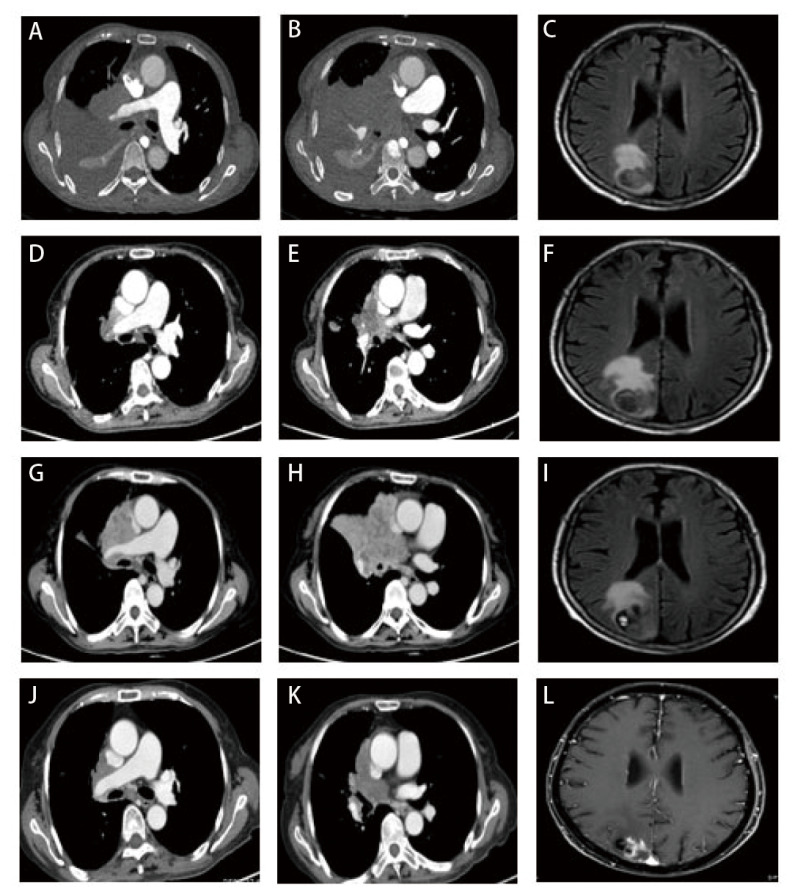
患者治疗过程中的典型影像学检查结果。 A-C：基线胸部CT和颅脑MRI（2024-03-10）；D-F：2个周期化疗联合免疫治疗后的胸部CT和颅脑MRI（2024-05-05）；G-I：首次病情进展时的胸部CT和颅脑MRI（2024.07.13）；J, K：二线双靶治疗后的胸部CT（2024.08.06）；L：二线双靶治疗、颅脑放疗后的颅脑MRI（2024.09.12）。

**图3:**
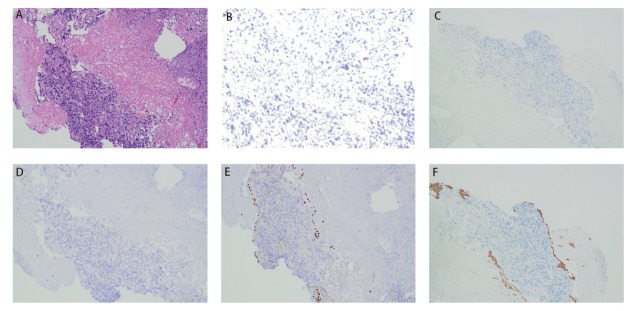
支气管镜活检病理。 A：HE染色（×100）；B：PD-L1免疫组化（×100）；C：TTF-1免疫组化（×100）；D：NapsinA免疫组化（×100）；E：p40免疫组化（×100）；F：CK5/6免疫组化（×100）。

患者行胸腔穿刺置管术，引流出3500 mL淡黄色清亮胸腔积液。胸水送检细胞学未查见癌细胞。电子支气管镜活检标本行下一代测序技术（next-generation sequencing, NGS）基因检测，结果显示*BRAF*基因NM_004333.6c.1455_1464delinsT（p.L485_T488delinsF）突变（第12外显子非移码缺失突变），丰度为54.31%；程序性死亡-配体1（programmed death-ligand 1, PD-L1）免疫组化：肿瘤细胞阳性比例评分（tumor proportion score, TPS）<1%。2024年3月18日起行一线化疗联合免疫治疗，方案为舒格利单抗1200 mg d1+白蛋白结合型紫杉醇200 mg d1,8（剂量按照125 mg/m²计算）+卡铂400 mg d1[血药浓度-时间曲线下面积（area under the curve, AUC）=5]，21天为1个周期。2个周期后复查CT示胸部病灶较前显著缩小，疗效评价为部分缓解（partial response, PR）（图2D、2E）。行颅脑MRI检查示病灶部分增大、部分缩小，增大者考虑合并瘤内出血（图2F）。2024年5月28日，患者拟行第4个周期治疗前出现发热、寒战，合并急性肾功能不全（肌酐：266 μmol/L），经肾内科抗感染及保肾治疗近1月后肾功能恢复正常，结合病史及治疗反应，患者肾功损伤倾向为感染相关性。2024年7月12日，患者再次因高热（峰值达40 ^o^C）、畏寒、胸闷等症状就诊，微生物学检查（血培养、痰培养）及感染指标（血清降钙素原、G试验、GM试验）均为阴性，予以左氧氟沙星联合美罗培南抗感染治疗，发热无明显改善，应用非甾体类抗炎药（non-steroidal anti-inflammatory drugs, NSAIDs）及激素治疗后体温明显下降，但停药后复发（体温>38.5^ o^C），考虑发热原因为阻塞性肺炎合并癌性发热。同期复查CT及MRI显示原发灶显著增大，脑转移灶增大，评估为疾病进展（progressive disease, PD）（图2G-2I）。患者一线免疫联合化疗的PFS为4个月。因持续发热、胸闷及消瘦，患者美国东部肿瘤协作组体能状态（Eastern Cooperative Oncology Group performance status, ECOG PS）评分降至2分，化疗耐受性差，患者及家属表示放弃继续化疗。通过查阅文献^[4,5]^发现针对*BRAF* V600突变的BRAF抑制剂（达拉非尼）和MEK抑制剂（曲美替尼）双靶治疗方案可能对携带*BRAF*非V600插入缺失突变的恶性肿瘤有效。与患者及家属充分沟通并签署知情同意书后于2024年7月20日起行达拉非尼联合曲美替尼方案（达拉非尼150 mg，口服，*bid*；曲美替尼2 mg，口服，*qd*）靶向治疗，同时进行了脑转移灶局部放疗，剂量为50 Gy/20 f。因患者在靶向治疗前已存在持续发热并长期应用NSAIDs退热治疗，靶向治疗1 d后，患者体温升高至40^ o^C，遂给予联合激素治疗，但体温仍在38.5^ o^C以上，将达拉非尼剂量下调至75 mg *bid*后体温降至正常。后尝试恢复原剂量（150 mg *bid*），但因发热症状反复，最终维持达拉非尼75 mg *bid*的减量方案。双靶向治疗后患者发热停止，一般状况好转，靶向治疗半个月后复查CT示胸部病灶显著缩小，评估疗效为PR（图2G、2K）。后患者继续双靶治疗，2024年9月复查胸部CT示肺原发灶持续缩小，颅脑MRI示脑转移瘤显著退缩（图2L），评效为PR。患者持续规律服药，至2024年11月5日患者再次出现发热、胸闷症状，复查胸部CT显示右肺肿瘤较前增大、胸腔积液增多，病情进展，二线双靶治疗的PFS为3.5个月。图4展示了患者临床诊断和治疗的时间线。本病例报告已获得患者家属知情同意。

**图4:**
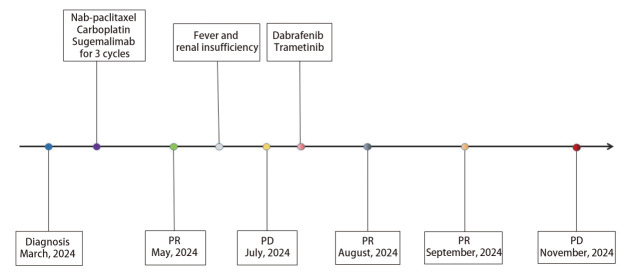
患者临床诊断和治疗的时间线

## 2 讨论

肺癌是我国及世界范围内发病率及死亡率均居第一位的恶性肿瘤，其中NSCLC占所有肺癌病理类型的80%以上，主要亚型包括腺癌、鳞癌和大细胞癌^[1]^。当肿瘤缺乏明确的腺癌或鳞癌形态学特征或免疫组化标志时，则归类为NSCC-NOS。本例患者因缺乏腺癌、鳞癌及神经内分泌癌的形态学及免疫表型特征，病理诊断为NSCC-NOS。根据美国国立综合癌症网络（National Comprehensive Cancer Network, NCCN）指南，NSCC-NOS与腺癌、大细胞癌同属非鳞NSCLC范畴，推荐进行基因检测以指导治疗决策。该患者NGS基因检测结果显示*BRAF*基因第12外显子非移码缺失突变，属于*BRAF*非V600突变，因此一线治疗方案参照无明确驱动基因的非鳞NSCLC诊疗规范。

*BRAF*突变在生物学上表现出显著的异质性。根据不同突变类型对*BRAF*激酶功能的影响、RAS依赖性以及作用机制的不同，可将其分为3类：激活型突变（I类和II类）和抑制型突变（III类）。其中，*BRAF* V600突变属于I类突变，其编码的蛋白以单体形式发挥作用，通常不与影响MAPK信号通路的其他致癌突变共存，这类患者能够从BRAF与MEK抑制剂的联合治疗中显著获益^[6,7]^。II类突变则形成不依赖RAS活性的功能性二聚体，理论上可能受益于MEK-ERK抑制剂或RAF（rapidly accelerated fibrosarcoma）二聚体抑制剂联合下游MEK/ERK抑制剂的治疗方案，但目前针对这类突变的治疗效果仍显著低于I类突变。III类突变表现为激酶活性缺失的异源二聚体，其功能依赖于RAS活性，采用上游激酶抑制剂联合下游MEK-ERK抑制剂的方案可能更具治疗潜力^[8-10]^。在临床研究方面，多队列II期BRF113928研究^[11,12]^已证实达拉非尼联合曲美替尼对*BRAF *V600E突变NSCLC患者中的显著疗效，ORR在初治和经治患者中分别达到63.9%和68.4%，中位PFS分别为14.6和10.2个月。基于此，美国食品药品监督管理局（Food and Drug Administration, FDA）和欧洲药品管理局（European Medicines Agency, EMA）批准该联合方案用于*BRAF* V600E突变晚期NSCLC患者的治疗。然而，对于*BRAF* V600非E突变的临床研究仍较为有限。法国AcSé试验^[13]^的NSCLC队列数据显示，4例接受维莫非尼单药治疗的*BRAF*非V600E突变患者的PFS分别为3.8个月（V600D）、5.9个月（V600M）以及2.1和6.8个月（2例V600K），提示部分*BRAF *V600非E突变可能仍对BRAF抑制剂具有一定敏感性。值得注意的是，尽管II类和III类*BRAF*突变约占所有*BRAF*突变病例的2/3，但目前尚缺乏有效的治疗方案，这一领域亟需加强研究。EURAF研究^[14]^显示，在6例*BRAF*非V600E突变患者中仅1例G596V突变患者对维莫非尼单药治疗产生部分反应。法国AcSé试验^[13]^进一步表明，在17例*BRAF*非V600突变患者（包括G469A、G466V、N581S、K601E、K601N、G466A、G469V和G596R突变）中，维莫非尼治疗的ORR为0%，中位PFS仅为1.8个月，中位生存期为5.2个月，显著低于*BRAF* V600E突变患者。这些数据提示，II类和III类*BRAF*突变虽然能够形成RAF二聚体并激活MAPK通路，但对现有BRAF抑制剂的反应较差，可能与其激酶活性依赖RAS或二聚化特性有关。

本例患者携带的*BRAF*非V600突变（p.L485_T488delinsF）定位于αC-螺旋与P环（磷酸结合环区）之间的关键功能域，其致病机制具有双重可能性。从分子结构角度分析，该突变可能通过两种途径影响激酶功能：一方面可能通过干扰腺苷三磷酸（adenosine triphosphate, ATP）结合口袋的立体构象，导致激酶活性部分保留，表现出类似II类突变的特征；另一方面则可能通过增强BRAF-丝氨酸-苏氨酸激酶1（raf-1 proto-oncogene, CRAF）异源二聚体形成，呈现类似III类突变的特性^[9,10]^。有体外实验证据^[5,15]^表明，结构相似的缺失突变（如p.L485_P490del）能够引起MEK/ERK信号通路的持续活化。关于*BRAF*插入缺失突变靶向治疗的临床数据较为有限。Zhang等^[5]^报道了1例携带*BRAF* p.L485-P490缺失突变的晚期恶性黑色素瘤患者，经达拉非尼联合曲美替尼二线治疗后达PR，且PFS超过18个月。另外，1例*BRAF* N486_P490缺失突变的晚期胰腺导管腺癌患者接受相同方案治疗8周后，肿瘤明显退缩，腹水减少，PFS达6个月，后患者死于严重急性呼吸综合征（severe acute respiratory syndrome, SRAS）冠状病毒感染^[4]^。这些跨瘤种个案报道和本病例观察到的PR（包括原发灶和颅内转移灶的退缩）均支持该方案对*BRAF*插入缺失突变可能具有的治疗价值。

本文报道了1例携带*BRAF* p.L485_T488delinsF插入缺失突变的晚期NSCLC患者的治疗经验。该患者在二线治疗中接受达拉非尼联合曲美替尼双靶方案后观察到显著的肿瘤退缩（PR），证实了该方案对这类罕见*BRAF*非V600突变患者的潜在治疗价值。值得注意的是，尽管治疗初期疗效显著，但患者的PFS相对较短（3.5个月），这一现象可能与该突变兼具II和III类突变的复杂生物学特性有关，同时也提示需要进一步优化治疗策略以延长疗效持续时间。本个案为*BRAF*非V600插入缺失突变NSCLC患者的临床诊疗提供了重要参考，并强调了对这类罕见突变开展更深入机制研究和更大样本临床验证的必要性，以期为患者制定更精准有效的治疗方案。
